# Beyond Hip Fracture: Orthopaedic Trauma in an Aging America

**DOI:** 10.1093/geroni/igaa057.851

**Published:** 2020-12-16

**Authors:** Lisa Reider, Joseph Levy, Andrew Pollak

**Affiliations:** 1 Health Policy and Management, Baltimore, Maryland, United States; 2 University of Maryland, Baltimore, Maryland, United States

## Abstract

Trauma related death and disability is common among working-age Americans, however the impact on older adults is consequential and increasing. Fractures are the most common traumatic injury diagnosis among Medicare beneficiaries, and though fragility fractures continue to be an important health problem, recent data indicate an increase in high-energy fractures. The purpose of this study was to produce national incidence estimates among US men and women ≥ 65 years using data from the 2003-2014 National Inpatient Sample (NIS). The study cohort included hospitalizations involving upper and/or lower extremity fractures which were further classified by mechanism as high or low energy using external cause of injury codes. Incidence was computed using survey weights provided by NIS, and population estimates from the Census Bureau. The incidence of high-energy fractures increased from 744.1/100,000 persons (95%CI: 681.1–807.1) in 2003 to 821.4/100,000 (95%CI: 795.0 – 874.8) in 2014 in women, and from 359.1/100,000 (95%CI: 331.4–386.8) to 408.2/100,000 (95%CI: 394.–809.2) in men. Over 80% were motor vehicle related. The greatest increase was among those ≥ 85 (1,856.4/100,000 to 2,126.3/100,000 in women; 1,069.1/100,000 to 1,215.1/100,000 in men). Simultaneously, the incidence of low-energy fractures declined: 748.4/100,000 (95%CI: 687.5–809.2) to 443.8/100,000 (95%CI: 423.5 -464.1) in women, and 310.6/100,000 (95%CI: 285 – 336.2) to 206.3/100,000 (95%CI: 196.5 - 216) in men. Results suggest that fractures commonly seen in younger adults will be seen more frequently in older age. It is therefore essential to establish treatment pathways to optimize outcomes for the growing number of injured older adults.

